# Supplementary material for: Complexome profiling of the Chlamydomonas psb28 mutant reveals TEF5 as an early PSII assembly factor
Source: Plant Cell. 2025 Mar 17;37(6):koaf055. doi: 10.1093/plcell/koaf055 (PMC12164590; doi:10.1093/plcell/koaf055)
Supplement: koaf055_Supplementary_Data [file koaf055_supplementary_data.zip › psb28_interactive.zip!/plots/intensity/qualityPlots/peptides/FHL3_Cre07.g352350.t1.2.html]

### Cre07.g352350.t1.2

Trivial name: FHL3  
SeqCoverage [median]: 9.00  
Mass: 114.69 kDa  
  
 

| Sequence | MissedCleavage | Mass [Da] | wtIntensity | muIntensity | Score |
| --- | --- | --- | --- | --- | --- |
| AIAAEGGVR | 0 | 842.46 | 0.00 | 399.83 | 89.75 |
| ALGTPLSFDELMR | 0 | 1448.73 | 375.05 | 0.00 | 70.10 |
| ALQALTQALLDR | 0 | 1311.75 | 414.64 | 0.00 | 62.30 |
| GQVAEQVR | 0 | 885.47 | 1236.28 | 1501.59 | 144.38 |
| IGGVLLAGAPGTGK | 0 | 1209.71 | 3118.00 | 5298.57 | 101.38 |
| IIYMPLPDALGR | 0 | 1357.74 | 1085.35 | 0.00 | 78.93 |
| SAVPAPGEVPNR | 0 | 1192.62 | 0.00 | 526.26 | 53.76 |
| VAATSEAASGADTLR | 0 | 1418.70 | 1476.91 | 323.35 | 95.09 |
|  |  |  | 7706.2 | 8049.6 |  |
